# Liquid biomarkers associate with TGF-β Type I receptor and hypoxia in kidney cancer

**DOI:** 10.1038/s41392-025-02404-7

**Published:** 2025-09-26

**Authors:** Pramod Mallikarjuna, Cemal Erdem, Ruben Ilundain Beorlegui, Anders Larsson, Börje Ljungberg, Masood Kamali-Moghaddam, Maréne Landström

**Affiliations:** 1 Department of Medical Biosciences, Umeå University, Umeå, Sweden; 2 Department of Medical Biosciences, Science for Life Laboratory, Umeå University, Umeå, Sweden; 3 Department of Medical Sciences, Uppsala University, Uppsala, Sweden; 4 Department of Diagnostics and Intervention, Umeå University, Umeå, Sweden; 5 Department of Immunology, Genetics and Pathology, Science for Life Laboratory, Uppsala University, Uppsala, Sweden

**Keywords:** Urological cancer, Biomarkers

**Dear Editor**

Renal cell carcinoma (RCC) accounts for 2.2% of all cancers diagnosed and 1.8% of all cancer-related deaths.^1^ Clear cell RCC (ccRCC), an aggressive subtype, makes up about 70% of all RCCs and is often linked to poor prognosis and familial syndromes like von Hippel-Lindau (VHL) disease.^2^ Most ccRCC cases are asymptomatic in early stages. Despite the immeasurable benefits of early cancer detection and the emergence of molecular biomarkers in precision medicine, specific markers for ccRCC remain scarce, where ccRCC heterogeneity poses challenges for developing new treatments.

In this study, we used proximity extension assay (PEA) to analyze blood samples from 134 ccRCC patients and 111 age- and gender-matched healthy donors. We identified a panel of seven proteins (ANXA1, ESM1, FGFBP1, MDK, METAP2, SDC1, and TFPI2) that can distinguish patients from controls with high diagnostic sensitivity and specificity. Moreover, by studying protein expressions in solid tumors from the same patients, we revealed associations between the panel biomarkers and proteins in the TGF-β and VHL-HIF signaling pathways and NT5E (5’-Nucleotidase Ecto), a membrane protein catalyzing the conversion of extracellular nucleotides.

We used the Olink Target 96 Oncology II PEA-panel (Olink Proteomics, Uppsala, Sweden) to measure plasma protein levels in patients and healthy controls (Fig. 1a). Blood plasma levels of 92 oncology-related proteins were measured across all samples. The dataset was corrected for patient age, where no significant effect observed for gender. Moreover, no inter-assay variation (technical bias) between the three PEA plates was detected. Of the 91 biomarkers that passed quality control checks, 80 proteins were significantly altered in ccRCC patients compared to controls, with 59 upregulated and 21 downregulated proteins (Fig. 1a-left).

**Fig. 1:**
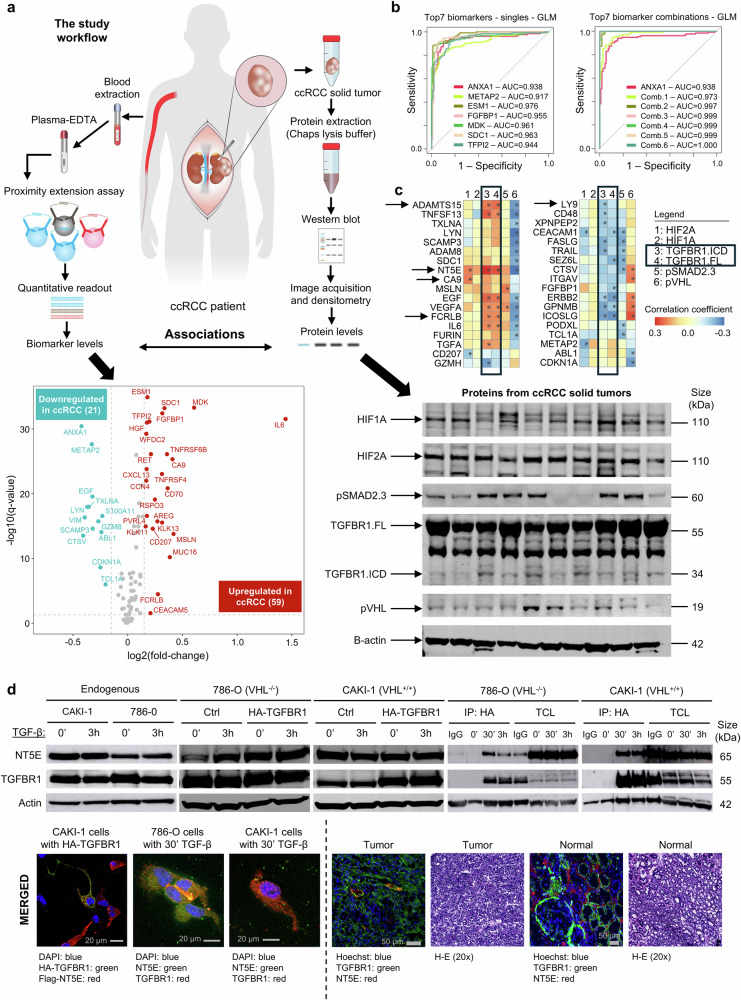
The plasma biomarkers are differentially expressed in ccRCC patients compared to healthy controls and are significantly correlated with TGF-β and HIF-α/pVHL pathway proteins in solid tumors. **a** The schematic illustration of the two parts of the study, which focuses on identifying novel biomarkers in the blood of patients with ccRCC. Additional analysis has been performed to investigate the associations between TGF-β and hypoxia pathway proteins from solid ccRCC tumors of the same patient cohort. The volcano plot summarizing the abundance of biomarkers significantly altered in ccRCC samples compared to healthy controls (two-sided, unpaired Wilcoxon Rank Sum test, *p*-values are FDR corrected. Only biomarkers with log2 fold-change greater than 0.15 or less than −0.15 with adjusted *p*-value < 0.05 are highlighted and labelled. Representative western blot image showing the protein bands of HIF1A, HIF2A, pSMAD2/3, TGFBR1-FL, TGFBR1-ICD, pVHL, and β-actin, in total protein extracted from 10 randomly chosen, different ccRCC solid tumor excisions. **b** The ROC curves for correctly classifying ccRCC patients and healthy controls, using gaussian linear models (GLM) and individual proteins one at a time (left) and in combination (right), show accuracy close to 100%. The red curves in both show the model performance when only ANXA1 data is used and each new curve shows the model performance with an additional biomarker, up to all seven. The model with all seven is a perfect classifier (AUC = 1). **c** Correlation matrix representing correlations between protein components of TGF-β and HIF-α/pVHL pathway proteins with serum biomarkers. pVHL was positively associated with CTSV, ICOSLG, ERBB2, GPNMB, and ITGAV, and negatively with ADAMTS15, SDC1, TNFSF13, NT5E, TXLNA, VEGFA, EGF, IL6, FCRLB, FURIN, SCAMP3, ADAM8, CDKN1A, and ABL1. For the TGF-β pathway, TGFBR1-Full length receptor (FL) and TGFBR1-intracellular domain (ICD), we report here, for the first time, positive associations with ADAMTS15 and FCRLB and negative associations with LY9, CD48, and XPNPEP2. The associations with SMAD2/3 -downstream effectors in the TGF-β canonical pathway- follow the observations for TGFBR1-FL and TGFBR1-ICD. * Denotes significance at *p*-values < 0.05, Spearman correlation, without investigation of contributions from potential confounders. The frame and arrows indicate some of the important associations described in the text. **d** Transient overexpression of TGFBR1 or stimulation with TGF-β1 induces NT5E in VHL-negative 786-O cells (immunoblots). Transient overexpression of HA-TGFBR1 in ccRCC cells, as indicated stimulated with TGF-β1 promotes binding of TGFBR1 to endogenous NT5E in two different ccRCC cell lines (immunoprecipitation). Transiently overexpressed TGFBR1 (HA-TGFBR1, green) and NT5E (red) proteins are colocalized in CAKI-1 cell membrane and cytoplasm (merged, yellow). DAPI (blue) stain represents nuclear regions. The scalebar in grey shows 20 μm. Endogenous TGFBR1 (red) and NT5E (green) are colocalized (merged, yellow) in the two ccRCC cell lines treated with TGF-β for 30 min, where the two proteins are likely to form protein-protein complexes. The scalebar in grey shows 20 μm (bottom left). Endogenous TGFBR1 (green) and NT5E (red) are colocalized (merged, yellow) in ccRCC tumors, while not in normal kidney cortex. DAPI and Hoechst (blue) stains represent nuclear regions. Hematoxylin-Eosin (H-E) show morphology. The scalebar in grey shows 50 μm (bottom right)

To evaluate the potential of these proteins in distinguishing tumor samples from control samples, we used the top 50 most significantly altered proteins to train a random forest (RF) model. The importance of each protein biomarker for classifying the data was inspected, with cross-validation model performances close to 100% (median = 100%, mean = 99.40%). Next, we identified the smallest subgroup of plasma biomarkers that could produce an accurate and precise diagnostic model for ccRCC. Based on the mean accuracy decrease metric of the RF models, we systematically included increasing number of proteins in a new RF model and observed that the top seven (ANXA1, ESM1, FGFBP1, MDK, METAP2, SDC1, and TFPI2) were sufficient to perfectly classify (AUC = 1) patients and healthy controls. Notably, Syndecan 1 (SDC1), a transmembrane glycoprotein, also shows strong positive correlation with all clinicopathological parameters (Spearman rank correlation *p*-value < 0.001). ESM1, FGFBP1 and MDK are important for angiogenesis and proliferation of cells, which possibly explain why they were found in this study. TFPI2, a serine protease inhibitor, is also known to be expressed in placenta, and has previously been demonstrated to be a highly specific serum biomarker for predicting ovarian cancer and recently, also to be elevated in plasma in kidney cancer patients.

To further test the performance of the identified seven-gene signature, we trained an elastic-net penalized logistic regression (ENLR) model. The ENLR model of the combined signature resulted in a perfect classifier (Fig. 1b-right), superior to any of the seven biomarkers individually (Fig. 1b-left). A random set of seven proteins and their combinations were not as significant as the selected top seven. The panel’s performance was internally validated using 10-fold cross-validation and held-out validation set. It is important to note that the observed AUC of 1.0 may be influenced by the limited cohort sizes and with a larger sample size this value would likely decrease due to increased variability.

We have previously reported that the transforming growth factor-β (TGF-β), VHL (tumor suppressor often mutated in ccRCC), and hypoxia (HIF-1α, HIF-2α) signaling pathways are interconnected in ccRCC,^3–5^ and that TGFBR1 contributes to the aggressiveness of ccRCC dependent on the VHL mutation status.^4^ Here, we explored the connections between these pathways by investigating the correlation of plasma protein levels and protein expression in solid tumors from the same cohort (Fig. 1a-western blots and Fig. 1c), We mainly focused on key proteins in the TGF-β and VHL/hypoxia pathways.

The analysis of the hypoxia pathway in ccRCC tumors showed that HIF-2A was positively associated with NT5E and CA9 (Fig. 1c). The identification of a correlation between the TGF-β pathway and NT5E is particularly interesting as NT5E is emerging as a crucial oncogenic factor in immunoregulation in cancer biology, with ongoing clinical studies targeting NT5E. Since we observed a strong positive correlation between TGFBR1 expression in solid tumor tissues and NT5E, we examined the endogenous basal protein levels in two ccRCC cell lines, and in response to TGF-β, and/or transient overexpression of HA-TGFBR1 (Fig. 1d). Notably, TGF-β stimulation of both 786-O and CAKI-1 cells caused an association of TGFBR1 and NT5E. Moreover, we investigated a possible association between transiently overexpressed TGFBR1 and NT5E in CAKI-1 cells using co-immunofluorescence, finding that the two proteins colocalized in the cell membrane and cytoplasm (Fig. 1d-bottom left). We furthermore demonstrated that TGFBR1 and NT5E colocalize in both cell lines when stimulated with TGF-β for 30 min (Fig. 1d-bottom left) as well as in tumor sections derived from patients with ccRCC, while not in kidney cortex tissue (normal) sections (Fig. 1d-bottom right). We concluded that TGF-β stimulation of ccRCC cells positively affects NT5E expression and that TGFBR1 and NT5E may form a protein-protein complex in the cell membrane and cytoplasm.

Overall, we applied statistical modeling to reduce 91 plasma proteins to a refined panel of seven ccRCC biomarkers that can discriminate between healthy controls and patients. Notably, many of these markers are secreted proteins, supporting their suitability as blood plasma biomarkers. Additionally, we analyzed solid tumor samples from the same patients to explore the connections between the signature proteins and the TGF-β and hypoxia/VHL signaling pathways, revealing several novel connections. These insights enhance our understanding of ccRCC tumor biology and pave the way for the diagnostic and prognostic potential of liquid biomarkers.

## Supplementary information

Supplementary Material

## Data Availability

Data used in the current work are available at 10.17044/scilifelab.28711088. The analyses scripts are available at https://github.com/malandstromlab/Biomarkers.
