# Supplementary material for: Liquid biomarkers associate with TGF-β Type I receptor and hypoxia in kidney cancer
Source: Signal Transduct Target Ther. 2025 Sep 26;10:309. doi: 10.1038/s41392-025-02404-7 (PMC12464240; doi:10.1038/s41392-025-02404-7)
Supplement: Supplementary file 1 — Supplementary Material [file 41392_2025_2404_MOESM1_ESM.docx]

Supplementary Materials for

**Liquid biomarkers associate with TGF-β Type I receptor and hypoxia in kidney cancer**

Pramod Mallikarjuna, Cemal Erdem, Ruben Ilundain Beorlegui, Anders Larsson, Börje Ljungberg, Masood Kamali-Moghaddam, Maréne Landström

Correspondence to: [marene.landstrom@umu.se](mailto:marene.landstrom@umu.se)

**This PDF file includes:**

Materials and Methods

Supplementary Text

Materials and Methods

**Patient and control participant recruitment:** A total of 142 ccRCC patients from northern Sweden who underwent nephrectomy (64 men and 78 women) with a median age of 66.50 years (range 32-87 years) were included in the study. The blood and solid tumor samples were collected only after obtaining written and informed consent from the patients. All patients were surgically treated between 2000 and 2009 and samples that passed the routine quality checks were considered. The control participants who donated blood for the study were recruited, comprising 60 men and 61 women with an age range between 21-79 years and a mean age of 51.02 years. We collected surplus EDTA plasma samples from healthy blood donors, and they were made unidentifiable by removing identifiable study subject information, except age in year and gender. The surplus sample use was approved by Uppsala ethical board (01/367).

**ccRCC patient follow-up and clinicopathological parameters:** In 142 patients, CT determined a mean tumor diameter of 70 mm (range 12–190 mm). Staging followed the 2009 TNM classification^1^ and was as follows: 58 patients in TNM stage I (40.8%), 23 patients in stage II (16.2%), 28 patients in stage III (19.7%), and 33 patients in stage IV (23.2%). The Fuhrman *et al*.^2^ grading system was used to grade the tumors, with the following grade distribution: 18 (12.7%) tumors were grade 1, 54 (38.0%) grade 2, 46 (32.4%) grade 3, and 24 (16.9%) grade 4. Patient follow-up performed using a scheduled program was employed for survival analysis. As of October 2022, 36 (25.4%) patients with ccRCC were alive without any indication of disease, 4 (2.8%) were alive with disease, 58 (40.8%) had died of ccRCC, and 44 (31.0%) had died of other causes.

**Protein extraction from blood and tumors:** A peripheral blood sample from the ccRCC patient cohort was collected before commencing any treatment. Similarly, blood was also collected from the healthy control group. The blood was collected in EDTA-coated tubes and centrifuged at 2000 x g for 3 hours to separate plasma. The plasma was stored at -80°C until use. Proteins from the frozen tumor tissues were extracted as previously described^3^. Briefly, a small piece of frozen tumor tissue of approximately 125 cubic millimeters was minced with a surgical knife, immediately mixed with 50 µl TRAPeze® 1X CHAPS Lysis Buffer (EMD Millipore, Billerica, MA, USA), and incubated for 30 min on ice with agitation. The mixture was then centrifuged at 14,000 rpm for 30 min, yielding a supernatant containing proteins. The total protein concentration was determined using the Thermo Scientific™ Pierce™ BCA™ Protein Assay Kit (Thermo Fisher Scientific, Waltham, MA, USA) following the manufacturer's instructions.

**PEA of blood plasma proteins:** To quantify the levels of putative oncogenic proteins in blood from ccRCC patients and healthy controls, we performed a proximity extension analysis assay (PEA) using the Olink multiplex Oncology II panel (Olink Proteomics, Uppsala, Sweden), which allows for the detection of 92 oncology-related protein biomarker candidates. The process and outcome were subjected to technical validation and quality control. The assay was carried out as previously described^4,5^. In short, 1 μl EDTA plasma sample was mixed with 3 μl incubation mix (containing 92 pairs of probes and 4 controls) in a 96-well microtiter plate. The mixture was incubated at 4°C overnight. Thereafter, 96 μl extension mix containing polymerase and PCR reagents was added. The mixture was incubated for 5 min at room temperature, 20 min at 50°C, followed by 17 cycles of DNA amplification. After completion, 2.8 μl of the amplification product was added to 7.2 μl detection mix in a new 96-well microtiter plate, from which 5 μl was transferred to a 96.96 Dynamic Array IFC (Fluidigm, South San Francisco, CA, USA), prepared and primed according to the manufacturer's instructions. The expression analysis was performed using the BioMark™ HD real-time PCR platform (Fluidigm, South San Francisco, CA, USA). The PEA real time PCR data were exported and normalized using Olink Wizard for GenEx software. A five-parameter log-logistic function was fitted to the standard curve measurements after outliers had been removed in a procedure based on the Grubbs test^6^. The limit of detection (LOD) was defined as the protein concentration in the fitted standard curve that corresponded to the PCR cycle threshold mCtblank − 2 sCtblank, where mCtblank and sCtblank denote the mean and standard deviation threshold cycle (Ct) values for the blank, respectively. The Ct values were normally distributed, allowing use of the t-test for comparison between groups. For further statistical analysis, the Olink-defined Normalized Protein eXpression (NPX) units were used (log2 scale). Full names and UniProtKB accession numbers for all 92 proteins are listed in Supplementary Data S13. Eight of the initial 142 patients and 10 of the initial 121 healthy samples were not quantifiable by the assay and thus excluded from the dataset for further analyses.

**Power analysis to determine sample size:** To distinguish between ccRCC patients and controls, power analysis was carried out using the R package MSstats^7^ using a false discovery rate of 0.05 and 92 proteins as features. The level for the power analysis of sample size was set to >0.8. The power analysis showed that 60 patients and 60 controls would be sufficient. Given this study’s sample size of 134 ccRCC cases and 111 controls, the present study has a statistical power of approximately 0.85 which is sufficient for the desired level of significance.

**Evaluation of technical bias:** We filtered the Olink dataset and excluded proteins with NPX levels below the limit of detection (LOD) in more than 60% of samples. Only the FADD protein measurements met this criterion, removed from the input data, and the remaining 91 proteins were retained for further analyses.

**Statistical analyses:** After collecting NPX data from the PEA experiments, principal component analysis (PCA) was performed to evaluate inter-assay variation (technical bias). Linear regression was then used to detect effects of age and gender on the same data, and the NPX values were adjusted if a significant effect was detected (p-value < 0.05). The correlation between pre- and post-adjusted NPX values for the proteins was calculated and plotted using the R software^8^ ggplot2^9^ (Figs. S1-S12). An initial test was performed to identify protein biomarkers (NPX data) significantly associated with either the disease (ccRCC) or control groups. The associations were estimated by a Wilcoxon signed-rank test. The R package corrPlot^10^ was used to calculate and present several correlations within the ccRCC cohort in this study. Firstly, the correlation between the biomarkers and clinicopathological parameters -cancer grade, cancer stage, and tumor diameter- were examined. Further, the correlations between the biomarkers were analyzed. Finally, the correlations between biomarkers and protein levels obtained from solid tumor were also analyzed. All the correlation values were obtained by executing a Spearman correlation test using the corrPlot, and a p-value less than 0.05 was considered significant.

**Multivariate analysis and Logistic Regression:** To evaluate the potential of the proteins in distinguishing tumor samples from control samples, we used the top 50 most significantly altered proteins from the previous Wilcoxon signed-rank test to train a random forest (RF) model using the R package randomForest^11^. The R package ggplot2^9^ was used to visualize the importance of each protein in classifying the data. Two RF plots were produced, one ranking the proteins according to predictive value and the reduction in accuracy when proteins are excluded from the model. The more accuracy is decreased when the protein is excluded, the more important the protein is for classification. The second plot shows the Gini coefficients. The mean decrease in the Gini coefficient measures how each variable contributes to the homogeneity of the nodes and leaves in the resulting random forest, with a higher value indicating the higher importance of the variable in the model. The predictive performance of the RF model was estimated by a 10-fold cross-validated RF model using a training set (80%) and a validation set (20%). The result from the validation test was plotted as a Receiver-Operator Characteristic curve (ROC). The R package “pROC^12^ was used to plot the ROC curve and for calculations of area under the curve (AUC) and other metrics, including optimal threshold, sensitivity, specificity, positive predictive value (PPV), and negative predictive value (NPV).

In order to identify a minimal diagnostic signature protein panel for ccRCC from the top 50 most significantly altered proteins, we used an elastic-net penalized logistic regression (ENLR) model in R package glmnet^13,14^. The ENLR model combines lasso and ridge regularization penalties to simultaneously perform regularization and variable selection. The data used for the model comprise a randomly selected training set composed of 80% of samples from patients and controls, and a validation set composed of the remaining 20% of samples. The penalization proportion (α) was defined by a grid search using 10-fold cross-validation and evaluated using sensitivity, specificity, AUC, and misclassification rates, a penalty of α = 0.2 was selected for the model’s penalty proportion, as it showed the highest accuracy and lowest error rate for misclassifications. To minimize the deviance of the fitted model, the tuning parameter λ was defined as the mean value of 100 iteratively computed lambda values. Regression coefficients were determined for each protein to evaluate the contribution of each protein toward the discrimination between disease and control samples. Following the 10-fold cross-validation, a subset of proteins that showed nonzero regression coefficients in all validation passes was selected for calculation of the final regression coefficients. A final set of seven ccRCC signature proteins was defined in a procedure in which an ROC curve was generated for the protein with the highest regression coefficient and then compared to an ROC curve produced with the addition of one more protein; the protein set was considered complete when adding more proteins did not produce further significant improvement in the ROC (Fig. S13).

**Analysis of tumor protein expression by immunoblotting (IB):** Candidate biomarkers identified by PEA and statistical analysis were evaluated in solid tumor samples by IB (Supplementary Data S14) as described^3^. Proteins were probed with IRDye® 800CW goat anti-rabbit or IRDye® 680RD goat anti-mouse antibodies (all LI-COR Biosciences) and imaged at 84 µm resolution using the Odyssey® CLx Infrared Imaging System. Band densitometry was analyzed with Image Studio™ Software v3.1 and normalized to β-actin. Antibodies are listed in Supplementary Data S15. Associations with TGF-β signaling and HIF-1α/HIF-2α were assessed using Spearman’s Rho (p<0.05). Figures use the synonyms TGFBR1-full length (FL) and TGFBR1-intracellular domain (ICD) (Supplementary Data S16).

**Cell culture and *in vitro* studies:** Caki-1 (VHL^+^/^+^, Sigma) and 786-O (VHL^-^/^-^, ATCC, authenticated in 2021) ccRCC cell lines were selected for their VHL status. Caki-1 cells were cultured in high-glucose DMEM with 10% FBS, 1% PEST, and 1% L-glutamine (all Sigma-Aldrich). 786-O cells were cultured and transfected with 5 µg HA-TGFBR1, NT5E-Flag (Origene #RC209568), or pcDNA 3.1 as described^3,15^. Starved cells were treated with 10 ng/mL TGF-β1 (R&D Systems) or left untreated as controls, then harvested at indicated time points (Fig. S14).

**Protein extraction, immunoblotting of HA-TGFBR1 and Flag-NT5E** performed as described^3,15^. Antibodies: Primary—HA (mouse, Cell Signaling #2367), NT5E (rabbit, Cell Signaling #13160), β-actin (mouse, Sigma-Aldrich #A1978); Secondary—anti-rabbit 800CW (LI-COR #926-32211), anti-mouse 680CW (LI-COR #926-68070).

**Immunoprecipitation (IP) of HA-TGFBR1 with NT5E:** Total cell lysates were prepared and IP performed with the indicated antibody, followed by immunoblotting as described^15^. Antibodies—Anti-HA (mouse, Cell Signaling), NT5E (rabbit, Cell Signaling); secondary—light chain–specific (Cell Signaling).

**Immunofluorescence and immunohistochemistry of endogenous TGBFR1 and NT5E** was performed in Caki-1 and 786-O cells as described^15^ using TGFBR1 (rabbit, V22, 1:150) and NT5E (mouse, Abcam #ab257309, 1:50) primary antibodies for 1 h at room temperature (RT), followed by AlexaFluor555 anti-rabbit (1:600) and AlexaFluor488 anti-mouse (1:600) secondary antibodies. Nuclei were stained with DAPI (Fig. S15). In frozen renal tissue, sections were fixed (ethanol:methanol 1:1, 10 min, −20°C), blocked (5% donkey serum, 50 min, RT), incubated overnight at 4°C with TGFBR1 (#PA5-98192, 1:100) and NT5E (#ab54217, 1:100), and detected with AlexaFluor555 anti-rabbit (1:300) and AlexaFluor488 anti-mouse (1:300). Nuclei were counterstained with Hoechst 33342 (1:500).

**References**

1. Motzer, R. J. *et al.* NCCN clinical practice guidelines in oncology: kidney cancer. *J Natl Compr Canc Netw* **7**, 618–630 (2009).

2. Fuhrman, S. A., Lasky, L. C. & Limas, C. Prognostic significance of morphologic parameters in renal cell carcinoma. *Am J Surg Pathol* **6**, 655–663 (1982).

3. Mallikarjuna, P., Raviprakash, T. S., Aripaka, K., Ljungberg, B. & Landström, M. Interactions between TGF-β type I receptor and hypoxia-inducible factor-α mediates a synergistic crosstalk leading to poor prognosis for patients with clear cell renal cell carcinoma. *Cell Cycle* **18**, 2141–2156 (2019).

4. Assarsson, E. *et al.* Homogenous 96-Plex PEA Immunoassay Exhibiting High Sensitivity, Specificity, and Excellent Scalability. *PLoS ONE* **9 (4)**, e95192 (2014).

5. Shen, Q. *et al.* A targeted proteomics approach reveals a serum protein signature as diagnostic biomarker for resectable gastric cancer. *EBioMedicine* **44**, 322–333 (2019).

6. Grubbs, F. E. Procedures for Detecting Outlying Observations in Samples. *Technometrics* **11 (1)**, 1–21 (1969).

7. Choi, M. *et al.* MSstats: an R package for statistical analysis of quantitative mass spectrometry-based proteomic experiments. *Bioinformatics* **30**, 2524–2526 (2014).

8. R Core Team. R: A Language and Environment for Statistical Computing. R Foundation for Statistical Computing (2024). ([https://www.R-project.org/](https://eur01.safelinks.protection.outlook.com/?url=https%3A%2F%2Fwww.r-project.org%2F&data=05%7C02%7Cmarene.landstrom%40umu.se%7C63eebdef33a54193c88a08ddde95b92d%7C5a4ba6f9f5314f329467398f19e69de4%7C0%7C0%7C638911456831826849%7CUnknown%7CTWFpbGZsb3d8eyJFbXB0eU1hcGkiOnRydWUsIlYiOiIwLjAuMDAwMCIsIlAiOiJXaW4zMiIsIkFOIjoiTWFpbCIsIldUIjoyfQ%3D%3D%7C0%7C%7C%7C&sdata=z0cqo2lEA7ITduREIiix9Rb7%2BEY4tunW2uVtYZms0LU%3D&reserved=0))

9. Wickham, H. *Ggplot2: Elegant Graphics for Data Analysis*. (Springer-Verlag New York, 2016).

10. Wei, T. & Simko, V. R Package ‘Corrplot’: Visualization of a Correlation Matrix. (2024). ([https://github.com/taiyun/corrplot](https://eur01.safelinks.protection.outlook.com/?url=https%3A%2F%2Fgithub.com%2Ftaiyun%2Fcorrplot&data=05%7C02%7Cmarene.landstrom%40umu.se%7C63eebdef33a54193c88a08ddde95b92d%7C5a4ba6f9f5314f329467398f19e69de4%7C0%7C0%7C638911456831847172%7CUnknown%7CTWFpbGZsb3d8eyJFbXB0eU1hcGkiOnRydWUsIlYiOiIwLjAuMDAwMCIsIlAiOiJXaW4zMiIsIkFOIjoiTWFpbCIsIldUIjoyfQ%3D%3D%7C0%7C%7C%7C&sdata=7Jc5%2BeZjLhmplQuA3Vvab01%2BEyQYdkW9mTu07ryvvlA%3D&reserved=0))

11. Liaw, A. & Wiener, M. Classification and Regression by randomForest. *R News* **2**, 18–22 (2002).

12. Robin, X. *et al.* pROC: an open-source package for R and S+ to analyze and compare ROC curves. *BMC Bioinformatics* **12**, 77 (2011).

13. Friedman, J., Hastie, T. & Tibshirani, R. Regularization Paths for Generalized Linear Models via Coordinate Descent. *J. Stat. Software* **33**, 1–22 (2010).

14. Tay, J. K., Narasimhan, B. & Hastie, T. Elastic Net Regularization Paths for All Generalized Linear Models. *J. Stat. Software* **106**, 1–31 (2023).

15. Mu, Y. *et al.,* The TβRI promotes migration and metastasis through thrombospondin 1 and ITGAV in prostate cancer cells**.** *Oncogene* **43,** 3321-3334 (2024)***.***
